# On the use of an optoacoustic and laser ultrasonic imaging system for assessing peripheral intravenous access

**DOI:** 10.1016/j.pacs.2017.01.002

**Published:** 2017-01-30

**Authors:** A.S. Bychkov, V.P. Zarubin, A.A. Karabutov, V.A. Simonova, E.B. Cherepetskaya

**Affiliations:** a Moscow Mining Institute, The National University of Science and Technology MISiS, 6 Leninskiy prospekt, Moscow 119049, Russia; b Faculty of Physics, M.V. Lomonosov Moscow State University, 1 Leninskiye Gory, Moscow 119991, Russia; c International Laser Center, M.V. Lomonosov Moscow State University, 1 Leninskiye Gory, Moscow 119991, Russia; d The Institute on Laser and Information Technologies of the Russian Academy of Sciences, 1 Svyatoozerskaya St., 140700 Shatura, Moscow Region, Russia

**Keywords:** Optoacoustic imaging, Laser ultrasonic imaging, Combined imaging, Real-time imaging, Needle tracking, Guidance

## Abstract

We describe a universal system for research in combined real-time optoacoustic (OA) and laser-ultrasonic (LU) imaging. The results of its testing on the task of needle insertion into the blood vessel model diagnostics are presented. In OA mode, where laser light is absorbed directly in the sample, the contents of blood vessel model is clearly visible. In LU mode, where the short ultrasonic probe pulse scattered on the sample is detected, the needle is clearly visible. The developed solution combining OA and LU imaging modalities due to the common detection system allowed real-time diagnostics of the position of medical needles (0.63 mm and 0.7 mm in diameter) inside blood vessel models (1.6 mm and 2.4 mm in diameter). Frame rate was 10 Hz. High longitudinal spatial resolution of the system − 0.1 mm − allows distinguishing the two walls of the vessel model and the position of the needle inside.

## 1 Introduction

Procedures involving needle insertion into biological tissues [1], blood vessels in particular [2], are widespread in medical treatments. These procedures are associated with a number of difficulties: blood vessels can be deep and/or poorly palpable, needle can miss or pierce through the selected vessel causing unnecessary damage and side effects which have to be treated later. In this context, it would be advantageous to have a device to locate the vessels that are most suitable for intravenous therapy procedures and to visualize the relative position of vessel and needle. This will not only facilitate the work of medical staff, but also will provide a way to specify the prerequisites necessary for injury reduction and new medical methods development.

The possibility of using traditional 3D ultrasound [1], [3], optoacoustic tomography [4] and combined optoacoustic & ultrasound imaging [5], [6], [7] for real-time monitoring of needle/catheter insertion into biological objects has been studied earlier. Moreover, to guide the tip of the catheter/needle more accurately, it can be made acoustically active [8]. Generally, it is recommended that needle placement should be performed under real-time ultrasound guidance if patients have difficult peripheral venous access [2]. Also, in literature much attention has been paid to ultrasound and optical methods for vein localization [9], [10].

Optoacoustic (OA) tomography is based on pulsed-laser excitation of acoustic signals in a medium with inhomogeneous optical absorption, and the subsequent detection of the acoustic signals with high temporal resolution [11], [12]. The absorption coefficient distribution in the sample can be reconstructed from the recorded signals. OA tomography provides high contrast images and has better spatial resolution in turbid media than purely optical imaging methods. Today, 2D and 3D optoacoustic imaging systems for breast cancer diagnostics [13], [14], which have the potential of being used in clinical practice, as well as real-time multi-spectral OA systems operating at several wavelengths [15], are being developed.

Laser ultrasonic (LU) diagnostics involves generation of broadband ultrasonic probe pulse by a laser pulse absorbed in a dedicated optoacoustic generator, and registration of scattered ultrasonic signals with high temporal resolution [11]. Spatial distribution of acoustical inhomogeneities in the sample is reconstructed from the recorded signals. Short duration of ultrasonic pulses enables high spatial resolution, which can be considerably higher than that of conventional ultrasound imaging. Both optoacoustic and laser ultrasonic methods use analogous detector arrays for registration of ultrasonic fields. It makes combination of these two modalities in one system possible. Such system provides high contrast images with high spatial resolution [16]. Combination of OA and LU imaging is possible if optoacoustic and laser ultrasound signals are registered by a common array of sensors, which enables the imaging in the same coordinate system.

Currently, research in the field of combined OA and LU imaging has been focused mostly on representative and well reproducible automated experiments, the development and testing of reliable specialized experimental setups, and complex computational and experimental studies of various applications [16], [17], [18], [19], [20], [21]. The demand for representative experimental tomographical studies of various objects is steadily increasing. It is of particular importance to develop and build experimental setups that will provide prerequisites for development of real-time compact imaging systems. Practical application of OA and LU imaging is unavoidably connected with experimental study of phenomena which cannot be taken into account by means of numerical simulation. That is why any experimental research in this area and the development of multi-functional experimental setups are of great importance with a view to developing theoretical models and engineering reliable combined OA and LU imaging systems.

## 2 Materials and methods

**Fig. 1:**
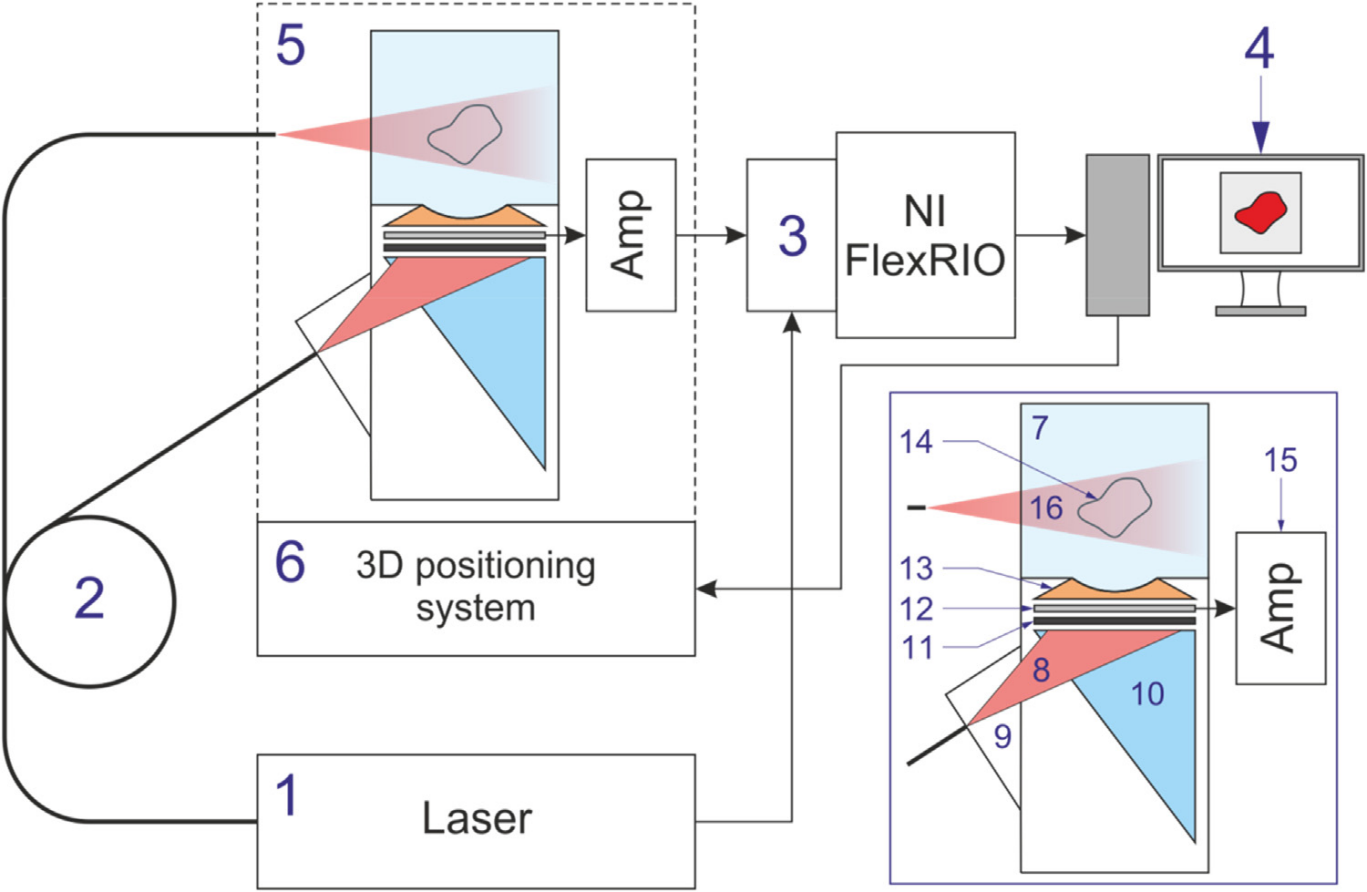
Schematic of the multi-channel system for combined OA and LU imaging: 1–laser; 2–fiber-optic laser radiation delivery system; 3–experimental data acquisition and processing system; 4–PC; 5–detector array; 6–3D positioning system. Inset shows a schematic of the detector array for combined OA and LU imaging: 7–tank with immersion liquid (distilled water); 8–laser radiation in LU mode; 9–optical system; 10–acoustic backing; 11–OA generator; 12–detector array; 13–acoustic lens; 14–sample under study; 15–amplifier; 16–laser radiation in OA mode.

**Fig. 2:**
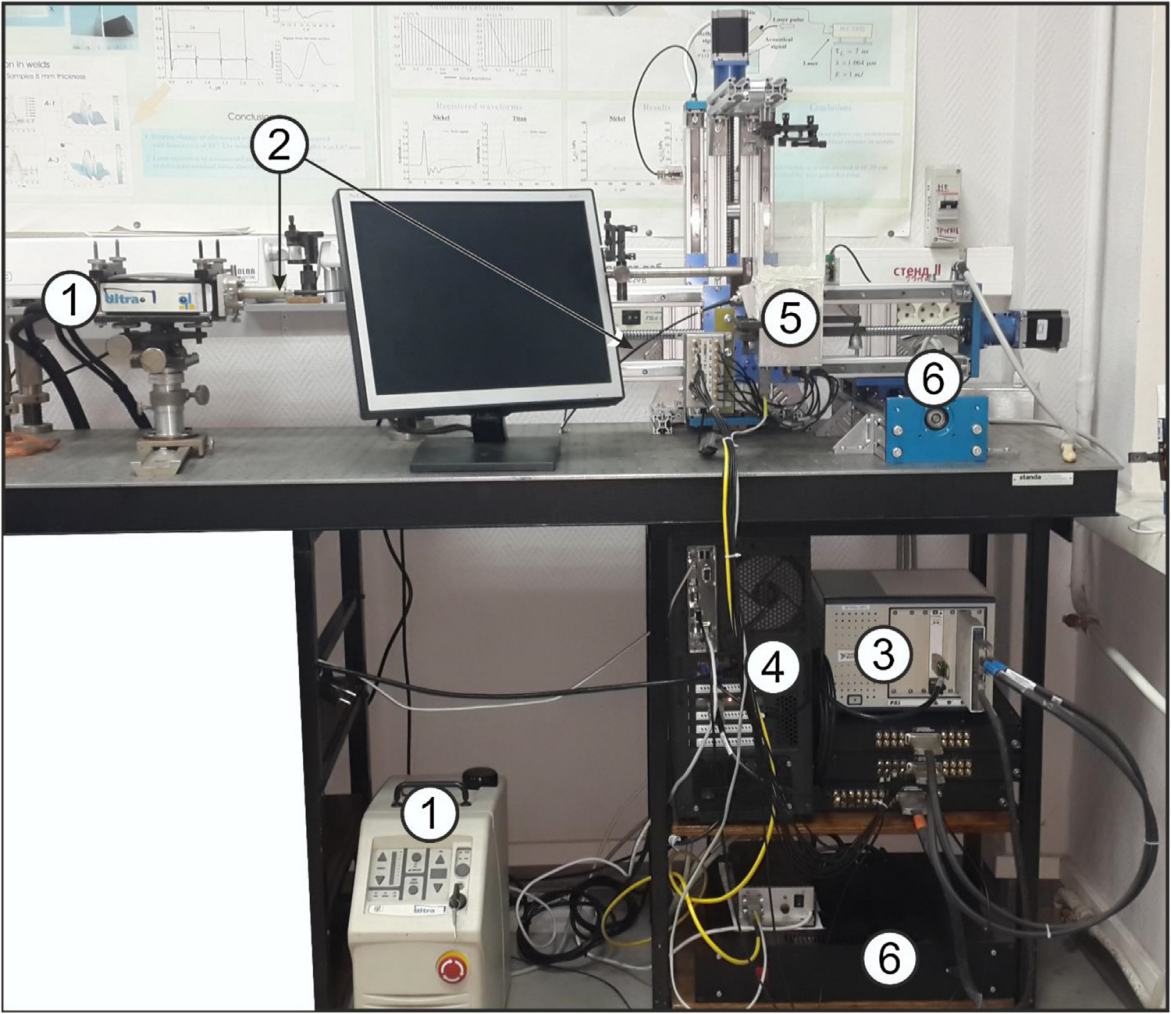
Photograph of the experimental setup: 1–laser with its power supply; 2–fiber-optic laser radiation delivery system in LU mode; 3–experimental data acquisition and processing system; 4–PC; 5–detector array; 6–3D positioning system.

A schematic of the multifunctional automated experimental setup for combined real-time OA and LU imaging is shown in Fig. 1. The inset shows a detailed schematic of the detector array (Links 2000, Russia) [16]. Fig. 2 shows the photograph of the experimental setup.

A Q-switched Nd:YAG laser (Quantel Ultra, Quantel, USA) is used as a laser source: λ = 1064 nm; pulse repetition rate is 20 Hz; pulse energy ∼10 mJ, pulse duration ∼10 ns. Through the fiber optic cable (1 mm core diameter, Optofiber, Russia), laser radiation is delivered either to the tested object (in OA mode) or to the OA generator in the detector array (in LU mode).

**Fig. 3:**
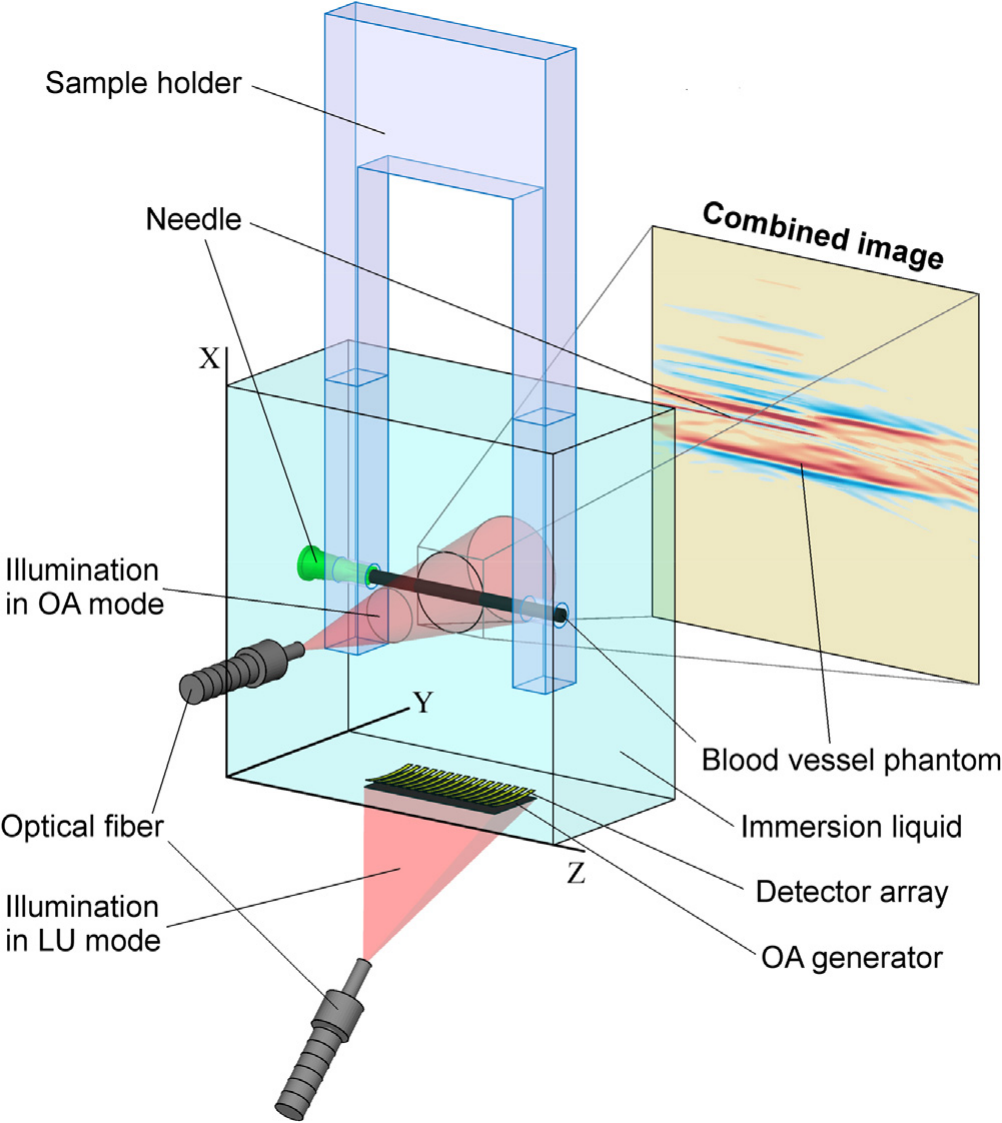
Schematic of experiments.

In OA mode, absorption of laser radiation causes heating and fast nonuniform expansion of the sample, which results in the generation of acoustic pulses. The pulses pass through a cylindrical acoustic focusing Plexiglas lens and are registered by a planar receiver array consisting of 16 piezoelectric PVDF transducers with a bandwidth from 1.6 to 9 MHz. Owing to wideband detectors, high longitudinal (depth) resolution is achieved: Δ*x* ≈ 0.1 mm. The acoustic lens forms image plane with thickness Δ*y* ≈ 0.4 mm at depth *f* ≈ 40.1 mm. Here we define experimental and theoretical resolutions as full widths at half maximum of the point spread function in the corresponding directions. Theoretical resolutions are determined using computer simulations of point spread function. Theoretical longitudinal resolution (along X axis, see Fig. 3) is 0.1 mm and is determined by the bandwidth of the receiving system. Theoretical lateral resolution (along Z axis) is 1 mm and is determined by the size of each receiver of the array in the corresponding direction.

In LU mode, laser radiation is absorbed in a built-in OA generator where a broadband acoustic probe pulse is generated. This optoacoustic generator is a plane-parallel plate made of polymeric material that has high laser radiation absorption efficiency, high thermal expansion coefficient, and acoustic impedance which matches the acoustic impedance of the material of the acoustic channel. Ultrasonic probe pulse generated in the OA generator passes through the acoustic lens. It is reflected and scattered from acoustic inhomogeneities in the object. Then scattered acoustic field passes through the lens to be registered by the detector array.

Analog electrical signal from the detectors is amplified and fed to a high-speed multichannel data acquisition and processing system based on NI FlexRIO architecture. The system contains a 32-channel analog-to-digital converter NI 5752 (National Instruments, USA) with a sampling rate of 50 MHz. It digitizes, stores, averages acquired data and transmits the averaged digital signals, via high speed communication lines (PCIe), to a personal computer (PC, Intel Core i7-4770 CPU @ 3.40 GHz, Intel, USA) in real time. The developed hardware-software solution enables the use of lasers with a pulse repetition rate up to 1 kHz.

Real-time GPU-based (Graphical Processing Unit) signal filtration is carried out in the range from 0.1 to 10 MHz. The filtered back-projection algorithm [22] is used to reconstruct OA and LU images in real time on GPU (NVIDIA GeForce GTX 770, NVIDIA, USA). It is taken into account that in LU mode the time of registration of scattered pulses is $t_{d}=\left(d+r\right)/c$, where *d* is the distance between the scatterer and the OA generator, *r* is the distance between the scatterer and the receiver, and *c* is the speed of sound in a medium.

To produce high-quality images it is necessary to position the sample under study relative to the detector array accurately. Therefore, the experimental setup is equipped with a custom-made automated 3D positioning system (Aketon, Russia) containing three motorized linear translators with step motors and optical limit switches. The detector array can be positioned in XY horizontal plane; the sample can be positioned vertically parallel to Z axis. The 3D positioning system is controlled by PC via USB.

Fig. 3 shows a general schematic of the experiment. A sample is fixed in a dedicated Plexiglas frame and placed into a tank filled with an immersion liquid (distilled water). Using the 3D positioning system, the object is placed in the focus of the detector array. In OA mode, laser radiation is delivered perpendicularly to the image plane.

Diagnostics of complex biological objects by means of combined OA and LU imaging should be preceded by theoretical and experimental studies of various model objects. During modelling studies, it is possible to conduct a series of repeatable experiments to improve diagnostics and imaging algorithms.

The experimental setup for combined OA and LU imaging has been tested on a number of model objects. Point spread functions were reconstructed for OA and LU modalities. Black polyethylene film 20 μm thick, which was illuminated from an optical fiber (600 μm core diameter, Optofiber, Russia), was used as a model of an OA point source. The end of optical fiber (200 μm core diameter, Optofiber, Russia) was used as a model of LU point scatterer. In addition, an image of a biological object (a liver piece 3 mm x 6 mm fixed on the end of optical fiber (200 μm core diameter)) was reconstructed. The experimental longitudinal (along X-axis, see Fig. 3) resolution was found to be 0.1 mm, the experimental lateral (along Z-axis) resolution was 1.1 mm.

Polymeric tubes (Raychman PBF, Raychman, Russia) 1.6 mm and 2.4 mm in diameter with a wall thickness of 0.2 mm and 0.25 mm, respectively, were used as blood vessel models. They were filled with diluted Indian ink (Gamma, Russia) at a ratio of 1:60 (effective optical attenuation coefficient $\mu_{eff}=\sqrt{3\mu_{a}\mu_{s}\left(1-g\right)}\approx 30\;\;{\text{cm}}^{-1}$, where *μ_a_* was absorption coefficient, *μ_s_* – scattering coefficient, *g* – anisotropy factor). According to [23] this value of attenuation coefficient corresponds to the blood (SatO_2_ = 50%, hct = 41%) attenuation coefficient at λ = 633 nm. Table 1 summarizes numerical estimates of acoustic parameters of the materials used.

## 3 Results and discussion

**Fig. 4:**
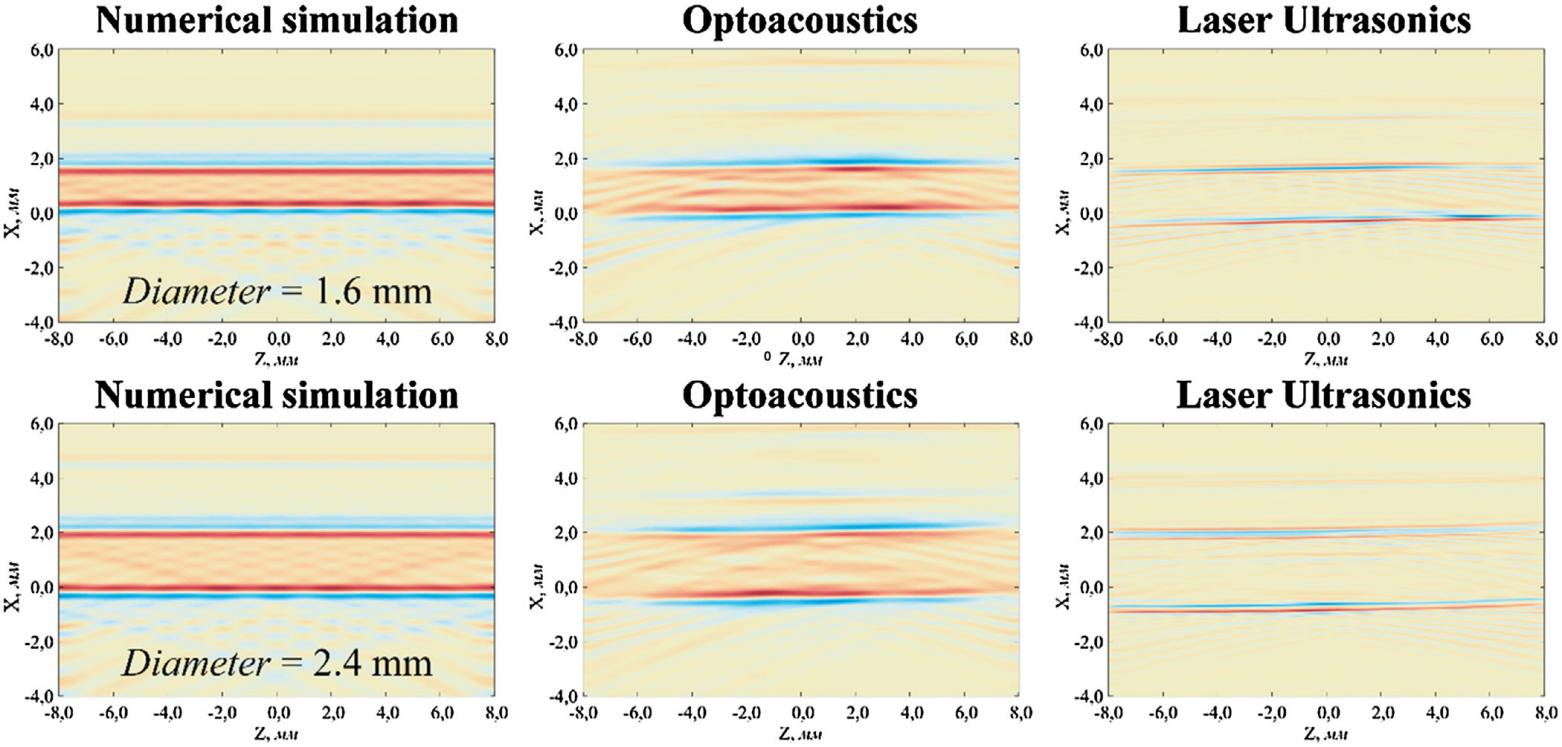
The results of numerical simulation (left), experimental OA images (center), and experimental LU images (right) of blood vessel models (polymeric tubes with an inner diameter of 1.6 mm (top) and 2.4 mm (bottom) filled with diluted Indian ink (1:60, optical attenuation coefficient 30 cm^−1^)).

The theoretical and experimental studies were exemplified by OA and LU images of blood vessel models with internal diameters of 1.6 mm and 2.4 mm and wall thickness of 0.2 mm and 0.25 mm, respectively, with no needle inside (Fig. 4).

During the experiments, a GPU-based real-time visualization system was used. The images presented below were reconstructed using MATLAB software package. In the images, the receiving detector array was on the line *x* = −40.1 mm. In LU mode the probe pulse comes from the bottom of the image (in the direction of X-axis). Color scale for the images was selected as follows. Since any image is a two-dimensional numerical array, the largest absolute value of intensity (*m*) was found, and then the color scale was uniformly distributed in the interval [−m,m]. The brightest white color corresponds to the value “zero”, red – to positive values, and blue color corresponds to negative values.

During numerical simulation of blood vessel models, the forward problem of optoacoustic tomography was solved:

(1) $$\left({\text{c}}^{2}\Delta -\frac{\partial^{2}}{\partial t^{2}}\right)p\left(\vec{r},t\right)=-\Gamma Q\left(\vec{r}\right)\frac{d\delta \left(t\right)}{dt}$$

where *c* was speed of sound, Δ – Laplace operator, $\Gamma =\frac{\beta c^{2}}{c_{p}}$ − Grüneisen parameter [22], $\beta =\frac{1}{V}{\left(\frac{\partial V}{\partial T}\right)}_{p}$ – volumetric thermal expansion coefficient, *c_p_* – specific heat capacity at constant pressure, δ(t) − Dirac delta function, $Q\left(\vec{r}\right)$ – spatial distribution of heat.

We considered a cylindrical source containing several radial fluid layers with different distributions of heat, sound speed, and density:

(2) $$\begin{array}{c} Q\left(r\right)=\{\begin{array}{cc} Q_{1} & r\leq r_{1}\\ Q_{n} & r_{n-1}<r\leq r_{n}\\ 0 & r>R \end{array}\\ c\left(r\right)=\{\begin{array}{cc} c_{1} & r\leq r_{1}\\ c_{n} & r_{n-1}<r\leq r_{n}\\ c & r>R \end{array}\\ \rho \left(r\right)=\{\begin{array}{cc} \rho_{1} & r\leq r_{1}\\ \rho_{n} & r_{n-1}<r\leq r_{n}\\ \rho & r>R \end{array} \end{array}$$

where *r* was the distance from the axis of the cylindrical source; *r*_1_, …, *r*_n_, …, *R* were the radii of the boundaries between the layers; *R* was the radius of the boundary between the source and immersion liquid.

Having solved the Eq. (1) for the Fourier transform of the pressure $\tilde{p}\left(\vec{r},\omega \right)=\overset{+\infty }{\underset{-\infty }{\int }}p\left(\vec{r},t\right)e^{-ı\omega t}dt$ for each layer, one would obtain the general solution:

(3) $$\begin{array}{cc} {\tilde{p}}_{1}\left(r,\omega \right)=A_{1}J_{0}\left(k_{1}r\right)-\frac{\iota \Gamma_{1}Q_{1}}{\omega } & r\leq r_{1}\\ {\tilde{p}}_{n}\left(r,\omega \right)=A_{n}J_{0}\left(k_{n}r\right)+B_{n}N_{0}\left(k_{n}r\right)-\frac{\iota \Gamma_{n}Q_{n}}{\omega } & r_{n-1}<r\leq r_{n}\\ \tilde{p}\left(r,\omega \right)=CH_{0}^{\left(2\right)}\left(kr\right) & r>R \end{array}$$

where *ω* would be angular frequency, $k=\frac{\omega }{c}$ – wavenumber, $J,N,H^{\left(2\right)}$ – Bessel, Neumann and Hankel functions, *A_n_*, *B_n_*, *C* – unknown coefficients.

For every boundary between two layers, two boundary conditions can be specified as follows: pressure *p* and vibrational acceleration $\vec{a}=-\frac{\nabla p}{\rho }$ should be continuous across the boundary. Coefficients *A_n_*, *B_n_*, *C* can be found by solving the resulting system of linear algebraic equations and consequently the temporal shape of the pressure signal at a given point in space can be determined.

The experimental OA images were in good agreement with the numerical simulation, which indicated that this model was suitable for real OA sources modelling and that the experimental setup was designed properly. In LU images the walls of blood vessel model were seen clearly. These results proved that the system could be used to locate a blood vessel model and determine its diameter, and that the developed model can be used for system optimization and image quality improvement.

**Fig. 5:**
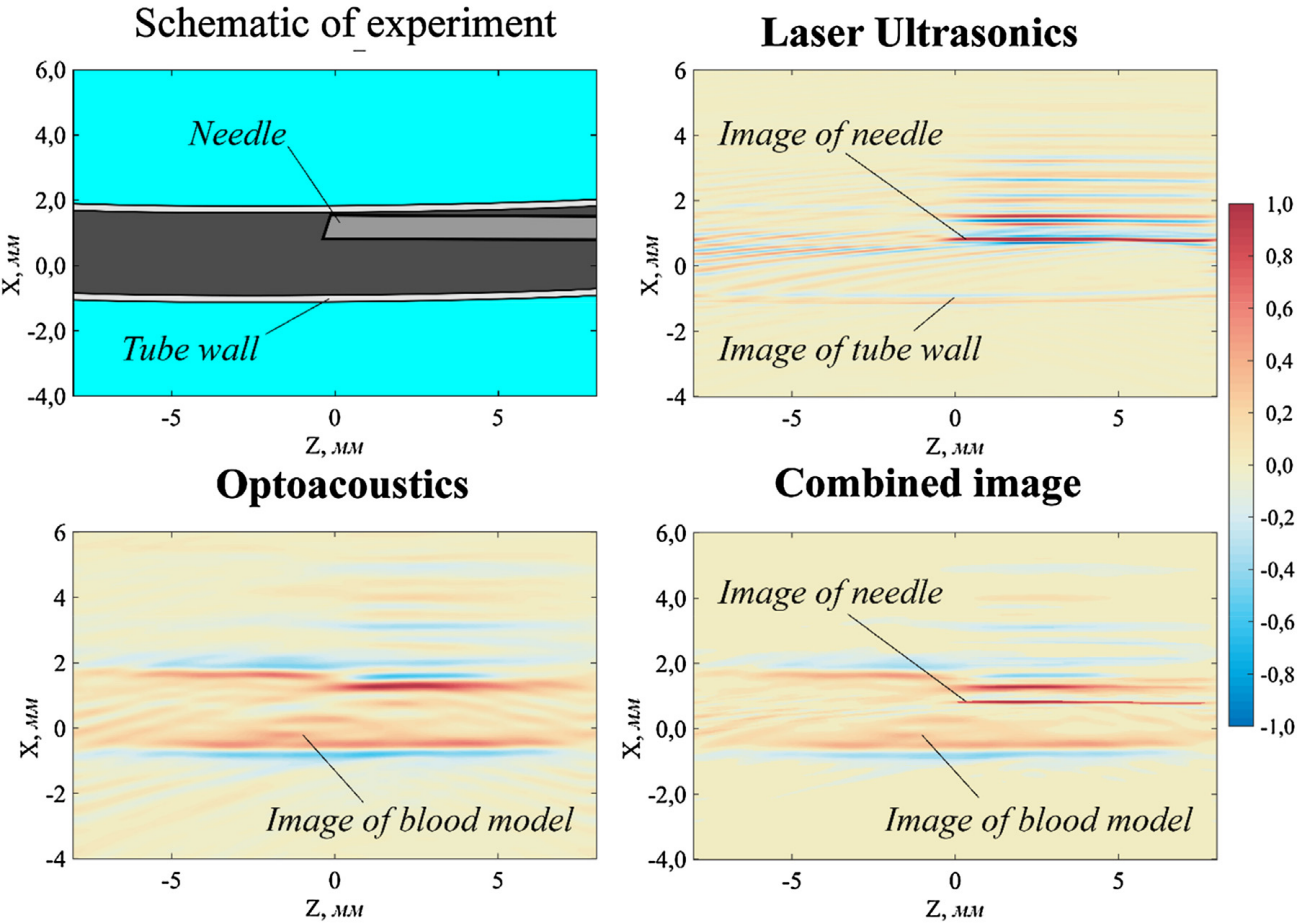
Experimental schematic (top left), LU image (top right), OA image (bottom left) and combined LU and OA image (bottom right) of needle 0.7 mm in diameter inside a polymeric tube 2.4 mm in diameter filled with diluted Indian ink (1:60, optical attenuation coefficient 30 cm^−1^).

Fig. 5 shows a schematic of the experiment and images of a needle (0.7 mm in diameter) inside a blood vessel model (2.4 mm in diameter) filled with diluted Indian ink.

The needle and tube wall are both visible in the LU image. The needle is an excellent acoustic reflector. It is seen much better because reflection of ultrasound from steel needle surface is much more efficient than from polymeric tube wall. Acoustic impedance of steel is substantially greater than acoustic impedances of immersion liquid (water) and tube material. In this case, the water-steel interface can be regarded as an acoustic hard boundary. Reflection of ultrasonic pulse occurs in phase and reflection coefficient is close to one. That is why the needle wall that is closer to the detector array is imaged as a red line in the LU image. Since almost all energy of the ultrasonic probe pulse is reflected back from needle wall, there is a region of acoustic shadow behind it (above it in the image). That is why the upper wall of the blood vessel model above the needle is not visible. The probe pulse is broadband and contains resonance frequencies of the needle. Therefore, it excites natural oscillations in the needle that can be seen in the LU images as periodic repetition of red and blue spots above the image of the needle wall.

In OA image, the vessel model wall is clearly visible where there is no needle. However, it is difficult to determine the position and shape of the needle accurately. Periodic repetition of red and blue spots in OA image suggests that the generation of ultrasound in the metal needle may be comparable to generation in diluted Indian ink. The wall of the vessel model behind the needle is not visible. This is probably due to ultrasound generation in the needle. It should be noted that in OA mode acoustic wave generated in the near-wall layer of the tube propagates not only outwards, but also inwards − towards the inside of the tube. The wave travelling inwards is reflected from the needle and can even excite natural oscillations in the latter.

**Fig. 6:**
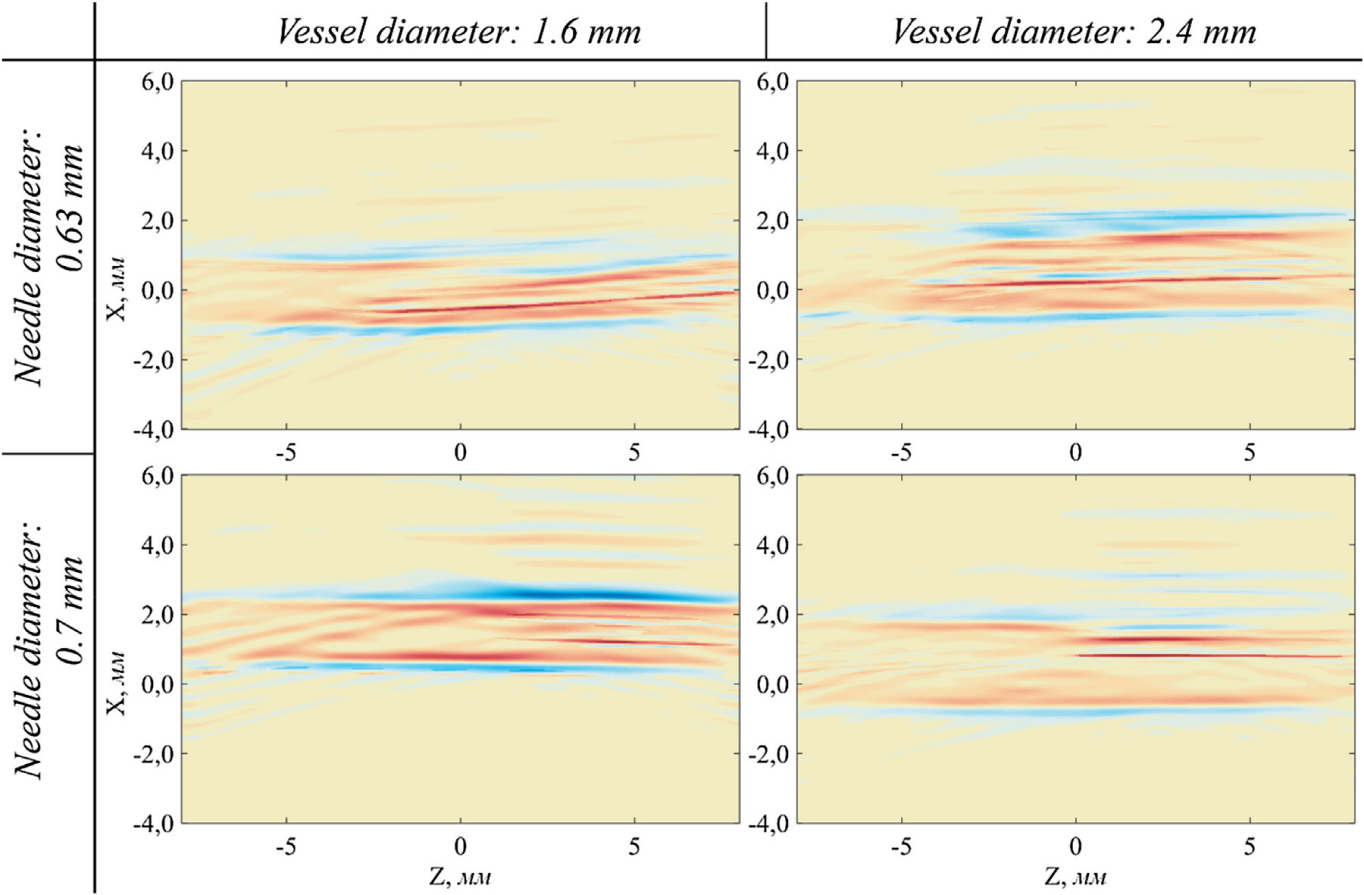
Experimental combined LU and OA images of needles (0.63 and 0.7 mm in diameter) inside polymeric tubes (1.6 and 2.4 mm in diameter) filled with diluted Indian ink (1:60, optical attenuation coefficient 30 cm^−1^).

Fig. 6 shows combined images of needles and blood vessel models with various diameters and relative position.

To form a combined image, LU and OA images are normalized to unity. The intensity values that are smaller than the threshold value, ¼ of the maximum, are zeroed. Then the images are summed and the sum is normalized to unity. Thus, the model vessel and the needle therein can be clearly seen. Importantly, OA and LU images can be combined accurately because these modalities share a common receiving system and both images are reconstructed in the same coordinate system.

These results suggest that the combined of optoacoustic and laser ultrasonic imaging system may allow reliable determination of the position of the needle in the blood vessel.

## 4 Conclusions

An experimental setup with an automated 3D positioning system has been constructed for combined 2D real-time optoacoustic and laser ultrasonic tomography (frame rate is 10 Hz). It is shown that in principle it is possible to locate a medical needle greater than 0.63 mm in diameter inside a blood vessel model with an outer diameter of 1.6 mm and 2.4 mm using combined OA and LU imaging. The needle is clearly seen in LU images and the blood vessel model is distinctly seen in OA images.

The combined OA and LU imaging system can be used not only for diagnostics of biological objects, but also to for examining the structure of various materials.

## Figures and Tables

**Table 1:** Numerical estimates of acoustic parameters of materials used in experiments.

Object	Sound velocity, ×10^3^ m/s	Density, ×10^3^ kg/m^3^	Acoustic impendence ×10^6^ Pa s/m
Water	1.5	1.0	1.5
Needle	5.9	7.9	46.6
Tube	2.1	0.9	1.9
